# The relationship between serum CA50, CA242, and SAA levels and clinical pathological characteristics and prognosis in patients with pancreatic cancer

**DOI:** 10.1515/med-2025-1304

**Published:** 2025-10-31

**Authors:** Zhicheng Yi, Fanhua Zhou, Xinkai Zhao

**Affiliations:** Department of Gastroenterology, The First Affiliated Hospital of Hainan Medical University, Haikou, 570100, China; Hainan Medical University First Clinical Medical College, Haikou, 570100, China; Department of Rheumatology and Immunology, The First Affiliated Hospital of Hainan Medical University, Haikou, 570100, China; Department of Gastroenterology, Hainan Medical University Affiliated Cancer Hospital, No. 9, Changbin West Fourth Street, Haikou, 570100, China

**Keywords:** pancreatic cancer, CA50, CA242, SAA, clinical pathological characteristics, prognosis

## Abstract

**Objective:**

This study aimed to investigate the relationship between serum carbohydrate antigen 50 (CA50), carbohydrate antigen 242 (CA242), and serum amyloid A (SAA) levels and clinical pathological features and prognosis in pancreatic cancer (PC) patients.

**Methods:**

A total of 163 PC patients were divided into a survival group (*n* = 43) and a deceased group (*n* = 120). Serum levels of CA50, CA242, and SAA were measured, and patients were categorized into high- and low-expression groups based on median values. Relationships between marker expression, clinical features, and prognosis were analyzed using Cox regression, receiver operating characteristic (ROC) analysis, and Kaplan–Meier curves.

**Results:**

Serum CA50, CA242, and SAA levels were significantly higher in the deceased group than in the survival group (all *P* < 0.001). High CA50 was linked to advanced TNM stage and distant metastasis; high CA242 to advanced TNM stage and lower differentiation; and high SAA to advanced TNM stage and distant metastasis (all *P* < 0.05). Multivariate Cox regression showed that advanced TNM stage (hazard ratio [HR] = 1.499, 95% confidence interval [CI] = 1.003–2.238, *P* = 0.048), distant metastasis (HR = 1.693, 95% CI = 1.157–2.478, *P* = 0.007), high CA50 (HR = 1.041, 95% CI = 1.019–1.064, *P* < 0.001), high CA242 (HR = 1.044, 95% CI = 1.018–1.070, *P* < 0.001), and high SAA (HR = 1.096, 95% CI = 1.044–1.151, *P* < 0.001) were independent risk factors for poor prognosis. ROC analysis showed that the combined detection of CA50, CA242, and SAA had the highest predictive value for poor prognosis (AUC = 0.989, sensitivity = 93.33%, specificity = 100%), which was significantly superior to single-marker detection (CA50: AUC = 0.872, sensitivity = 78.33%, specificity = 88.37%; CA242: AUC = 0.905, sensitivity = 74.17%, specificity = 88.37%; SAA: AUC = 0.871, sensitivity = 80.00%, specificity = 83.72%; all *P* < 0.001 vs combination). Kaplan–Meier curves revealed higher mortality risk in high-expression groups.

**Conclusion:**

Serum CA50, CA242, and SAA levels are closely associated with PC patients’ clinical features and prognosis. Their combined detection is a valuable tool for assessing poor prognosis in PC.

## 1 Introduction

Pancreatic cancer (PC) is a common malignant tumor of the digestive tract, with its incidence rate showing an upward trend year by year, making it the fourth leading cause of cancer-related deaths globally [1]. Due to its highly malignant nature and insidious onset, early diagnosis is extremely challenging, with most patients already having developed metastases by the time of diagnosis, resulting in an overall five-year survival rate of approximately 5% and a very poor prognosis [2,3]. Additionally, due to the non-specific nature of early symptoms and the lack of effective early diagnostic tools, most patients are diagnosed at an advanced stage, losing the opportunity for curative surgical treatment [3]. Therefore, early diagnosis holds great significance for the monitoring of PC progression and the assessment of its prognosis.

Current diagnostic methods have limitations. Multi-detector computed tomography (CT), although widely used as a preoperative examination in clinical practice and capable of providing good spatial resolution and anatomical coverage, still faces difficulties in accurately identifying early lesions [4,5]. Serum carbohydrate antigen 19-9 (CA19-9), while useful for monitoring treatment response and recurrence, is not suitable for early diagnosis due to its low sensitivity and specificity, and it may also be elevated in benign diseases such as chronic pancreatitis [6,7].

Recent studies have achieved new breakthroughs. A metabolomics study constructed a metabolite panel containing 16-hydroxypalmitic acid, phenylalanine, and norleucine. The area under the curve (AUC) of this panel for distinguishing resectable PC from healthy controls reached 0.942, and when combined with CA19-9, the AUC increased to 0.968 [8]. A radiomics model based on CT had an accuracy of 97.7%, a sensitivity of 97.6%, and a specificity of 97.8% in differentiating early-stage from late-stage pancreatic ductal adenocarcinoma (PDAC) [9]. Serum exosomal hsa-let-7f-5p was significantly upregulated in metastatic PC and could serve as a non-invasive diagnostic biomarker [10]. Additionally, emodin-conjugated PEGylated Fe₃O₄ nanoparticles enabled dual-modal imaging and targeted therapy for PC [11].

Against this backdrop, exploring tumor markers with high sensitivity and specificity is of great importance for monitoring the progression of PC and evaluating its prognosis. Carbohydrate antigen 50 (CA50) is a high-molecular-weight glycoprotein antigen widely present in various malignant tumor tissues, including PC, cervical cancer, and colorectal cancer (CRC) [12,13,14]. Studies have shown that serum CA50 levels in PC patients are significantly higher than those in benign tumor groups and healthy controls, suggesting its potential as an auxiliary indicator for the early diagnosis of PDAC [12]. Carbohydrate antigen 242 (CA242) is a salivary acidified carbohydrate antigen. Elevated serum CA242 levels have been clinically utilized as a diagnostic biomarker for various cancers, including PC, CRC, and other cancers [15,16]. Compared with other tumor markers, CA242 exhibits higher specificity in the early diagnosis of PC and its differential diagnosis from benign pancreatic diseases [17,18]. Serum amyloid A (SAA) is an acute-phase reactant protein primarily synthesized by the liver and significantly elevated in inflammatory disease states [19]. Recent studies have shown that chronic infection and inflammation, particularly the biosynthesis and secretion of pro-inflammatory cytokines, are closely associated with the development and progression of various cancers [20]. Multiple studies have found that SAA is upregulated in the serum of patients with various malignant tumors, such as gastric cancer, cervical cancer, and ovarian cancer, and its levels are closely associated with tumor occurrence, progression, and prognosis [21,22]. Additionally, it has been reported that SAA levels are associated with overall survival, progression-free survival, and treatment response in advanced PC patients receiving chemotherapy [23].

However, current research on the application of combined detection of serum CA50, CA242, and SAA in PC remains limited. Given the high heterogeneity of PC, a single tumor marker cannot meet the diagnostic needs of all cases, and both sensitivity and specificity are limited. Therefore, combined detection of multiple tumor markers may improve the accuracy of PC diagnosis and prognosis assessment. This study aims to investigate the relationship between serum CA50, CA242, and SAA levels and the clinical pathological characteristics and prognosis of PC patients, providing new insights and methods for the clinical diagnosis and treatment of PC.

## 2 Materials and methods

### 2.1 Study population

This retrospective study analyzed 220 patients with PC who visited our hospital between January 2021 and January 2023. After strict screening based on inclusion and exclusion criteria, 163 PC patients were ultimately included as the study population. Based on the prognosis of PC patients, they were divided into a survival group (*n* = 43) and a deceased group (*n* = 120).

### 2.2 Inclusion criteria

Inclusion criteria are as follows: (1) confirmed diagnosis of PC based on imaging and histopathological examination; (2) newly diagnosed and has not received chemotherapy or radiotherapy; (3) complete clinical and pathological data and postoperative follow-up data, complete postoperative 2-year follow-up data (including clear prognostic outcomes: survival to follow-up endpoint or death due to PC), and no missing key indicators (serum CA50, CA242, SAA levels); (4) age ≥18 years; and (5) the patient or their family members have been informed and have signed the informed consent form.

### 2.3 Exclusion criteria

Exclusion criteria are as follows: (1) presence of malignant tumors in other sites; (2) severe damage to vital organs such as the heart, brain, liver, or kidneys; (3) received any anticancer treatment prior to hospitalization; (4) pregnant or lactating women; (5) incomplete clinical data; and (6) inability to complete 2-year follow-up (e.g., expected loss to follow-up, refusal to commit to regular follow-up).

### 2.4 Data collection

Collect all clinical baseline and pathological data for PC patients, including age, gender, drinking history, smoking history, tumor location (head, body and tail), TNM stage (Stage I–II, Stage III–IV), tumor differentiation grade (high, moderate, low), lymph node metastasis (yes, no), distant metastasis (yes, no), serum CA50, CA242, and SAA levels, etc. Collect 5 mL of fasting morning blood from the elbow vein of all PC patients upon admission, place it in a collection tube, allow it to clot naturally at room temperature for 30 min, then centrifuge at 2,000 rpm for 20 min after the blood has clotted. Collect the upper serum layer and store it at −80°C for future use. CA50 and CA242 levels were measured using the German Roche Cobas E601 fully automated electrochemiluminescence analyzer. The serum level of SAA was determined using a commercial Kit for SAA protein assay (Ningbo Purebio Biotechnology Co, Ltd) and Automatic Analyzer H7180ID following the manufacturer’s instructions.

### 2.5 Follow-up and prognosis assessment

PC patients were followed up for 2 years after discharge through outpatient visits or telephone calls every 3 months, with the follow-up period ending in January 2025. Death was defined as a poor prognosis, and the prognosis of patients over 2 years was statistically analyzed.

### 2.6 Statistical analysis

Data statistical analysis and graphing were performed using GraphPad Prism 9.5.0 software (GraphPad Software Inc., San Diego, CA, USA) and SPSS 21.0 statistical software (SPSS, Inc., Chicago, IL, USA). The Shapiro–Wilk test was used to assess normality of distribution. Normally distributed continuous variables were expressed as mean ± standard deviation, and independent samples *t*-tests were used to compare between groups. Count data were expressed as counts and percentages, and chi-square tests were used for comparison. Univariate Cox regression was performed first. Variables with a *P* value of <0.05 in the univariate analysis were subsequently included in the multivariate Cox regression model to identify independent prognostic factors. Prior to constructing the multivariate model, the variance inflation factor (VIF) was used to assess multicollinearity among the biomarkers CA50, CA242, and SAA. The VIF values for CA50, CA242, and SAA were 1.258, 1.182, and 1.197, respectively, all well below the common threshold of 5 (or 10), indicating the absence of severe multicollinearity and their suitability for inclusion in the same multivariate model. Kaplan–Meier analysis and log-rank tests were used to compare distributions and plot survival curves for PC patients. Receiver operating characteristic (ROC) curves were used to assess the predictive value of serum CA50, CA242, and SAA for poor prognosis in PC patients. *P* values were obtained from two-sided tests, with *P* < 0.05 indicating statistically significant differences.


**Informed consent:** All participants were informed of the study objectives and provided written informed consent.
**Ethical approval:** The study was approved by The First Affiliated Hospital of Hainan Medical University, with informed consent requirements waived given the retrospective design. All procedures adhered to Helsinki Declaration principles and institutional regulations.

## 3 Results

### 3.1 Clinical baseline characteristics of the enrolled population

This study included 163 PC patients as subjects and divided them into a survival group (*n* = 43) and a deceased group (*n* = 120) based on their prognosis. As shown in Table 1, no significant differences in age, gender, drinking history, smoking history, or tumor location between the two groups (all *P* > 0.05). Additionally, the deceased group had more advanced TNM stage, lower differentiation, and higher rates of distant metastasis and lymph node metastasis compared with the survival group (all *P* < 0.05).

**Table 1:** Clinical baseline information of the enrolled population

	Survival (*n* = 43)	Deceased (*n* = 120)	*t*/*χ* ^2^	*P*
**Age (years)**	58.93 ± 6.59	60.18 ± 7.22	0.992	0.323
**Gender (** ***n*** **, %)**				
Male	23 (53.49%)	68 (56.67%)	0.129	0.719
Female	20 (46.51%)	52 (43.33%)		
**Drinking history (** ***n*** **, %)**	18 (41.86%)	52 (43.33%)	0.028	0.867
**Smoking history (** ***n*** **, %)**	14 (32.56%)	43 (35.83%)	0.149	0.699
**Tumor location (** ***n*** **, %)**				
Head	28 (65.12%)	82 (68.33%)	0.149	0.699
Body and tail	15 (34.88%)	38 (31.67%)		
**TNM stage (** ***n*** **, %)**				
Stage I–II	26 (60.47%)	40 (33.33%)	9.671	0.002
Stage III–ⅠV	17 (39.53%)	80 (66.67%)		
**Degree of differentiation (** ***n*** **, %)**				
Highly and moderately differentiated	2 8 (65.12%)	47 (39.17%)	8.581	0.003
Low differentiation	15 (34.88%)	73 (60.83%)		
**Lymph node metastasis (** ***n*** **, %)**				
Yes	13 (30.23%)	63 (52.50%)	6.307	0.012
No	30 (69.77%)	57 (47.50%)		
**Distant metastasis (** ***n*** **, %)**				
Yes	11 (25.58%)	58 (48.33%)	6.713	0.010
No	32 (74.42%)	62 (51.67%)		

Data are presented as *n* (%) or mean ± standard deviation. Categorical variables were compared using the Chi-square test. Continuous variables were compared using the independent samples *t*-test. *P* values were derived from two-sided tests, and a *P* value of <0.05 was considered statistically significant.

### 3.2 Comparison of serum CA50, CA242, and SAA levels in PC patients with different prognoses

**Figure 1:**
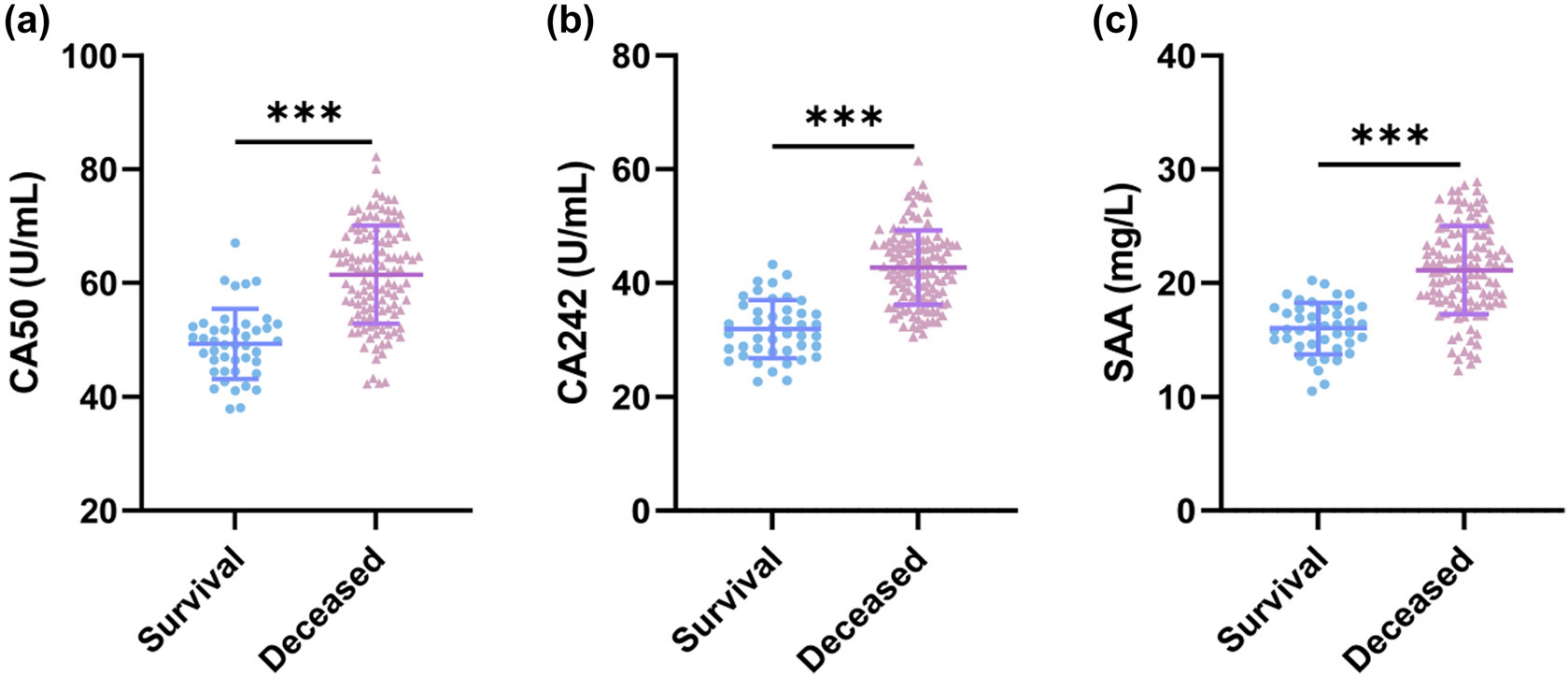
Comparison of serum CA50, CA242, and SAA levels in PC patients with different prognoses. (a) Comparison of serum CA50 levels; (b) Comparison of serum CA242 levels; (c) Comparison of serum SAA levels. The Shapiro-Wilk test was used to assess the normality of the distribution. The data in the figure meet the criteria for normality and are presented as mean ± standard deviation. An independent samples *t*-test was performed. *** indicates *P* < 0.001.

We compared serum CA50, CA242, and SAA levels in PC patients with different prognoses. The results showed that compared with the survival group, serum CA50, CA242, and SAA levels were significantly elevated in the deceased group (all *P* < 0.001) (Figure 1). These results indicate that serum CA50, CA242, and SAA levels were significantly higher in PC patients with poor prognosis than in those with good prognosis.

### 3.3 Relationship between serum CA50, CA242, and SAA levels in PC patients and clinical pathological characteristics

Patients were stratified into high- and low-expression groups for CA50, CA242, and SAA using their median values as cutoffs (CA50: 57.80 U/mL; CA242: 40.00 U/mL; SAA: 19.20 mg/L) to explore the association between marker levels and clinical pathological characteristics (Tables 2–4). High CA50 and SAA were both associated with advanced TNM stage and higher distant metastasis rate (all *P* < 0.05), while high CA242 was linked to advanced TNM stage and lower tumor differentiation (both *P* < 0.05). No significant associations were found between the three markers and age, gender, drinking history, smoking history, tumor location, or lymph node metastasis (all *P* > 0.05).

**Table 2:** Relationship between serum CA50 levels and clinical pathological characteristics in PC patients

	Low CA50 (*n* = 81)	High CA50 (*n* = 82)	t*/χ* ^2^	*P*
**Age (years)**	59.98 ± 7.032	59.72 ± 7.124	0.231	0.818
**Gender (** ***n*** **, %)**				
Male	44 (54.32%)	47 (57.32%)	0.148	0.700
Female	37 (45.68%)	35 (42.68%)		
**Drinking history (** ***n*** **, %)**	33 (40.74%)	37 (45.12%)	0.319	0.572
**Smoking history (** ***n*** **, %)**	28 (34.57%)	29 (35.37%)	0.011	0.915
**Tumor location (** ***n*** **, %)**				
Head	57 (70.37%)	53 (64.63%)	0.611	0.434
Body and tail	24 (29.63%)	29 (35.37%)		
**TNM stage (** ***n*** **, %)**				
Stage I–II	41 (50.62%)	25 (30.49%)	6.852	0.009
Stage III–ⅠV	40 (49.38%)	57 (69.51%)		
**Degree of differentiation (** ***n*** **, %)**				
Highly and moderately differentiated	41 (50.62%)	34 (41.46%)	1.375	0.241
Low differentiation	40 (49.38%)	48 (58.54%)		
**Lymph node metastasis (** ***n*** **, %)**				
Yes	34 (41.98%)	42 (51.22%)	1.399	0.237
No	47 (58.02%)	40 (48.78%)		
**Distant metastasis (** ***n*** **, %)**				
Yes	26 (32.10%)	43 (52.44%)	6.906	0.009
No	55 (67.90%)	39 (47.56%)		

Data are presented as *n* (%) or mean ± standard deviation. Categorical variables were compared using the Chi-square test. Continuous variables were compared using the independent samples *t*-test. *P* values were derived from two-sided tests, and a *P* value of <0.05 was considered statistically significant.

**Table 3:** Relationship between serum CA242 levels and clinical pathological characteristics in PC patients

	Low CA242 (*n* = 81)	High CA242 (*n* = 82)	*t*/*χ* ^2^	*P*
**Age (years)**	59.36 ± 6.45	60.33 ± 7.62	0.878	0.381
**Gender (** ***n*** **, %)**				
Male	43 (53.09%)	48 (58.54%)	0.491	0.484
Female	38 (46.91%)	34 (41.46%)		
**Drinking history (** ***n*** **, %)**	32 (39.51%)	38 (46.34%)	0.777	0.378
**Smoking history (** ***n*** **, %)**	33 (40.74%)	24 (29.27%)	2.358	0.125
**Tumor location (** ***n*** **, %)**				
Head	52 (64.20%)	58 (70.73%)	0.793	0.373
Body and tail	29 (35.80%)	24 (29.27%)		
**TNM stage (** ***n*** **, %)**				
Stage I–II	40 (49.38%)	26 (31.71%)	5.283	0.022
Stage III–ⅠV	41 (50.62%)	56 (68.29%)		
**Degree of differentiation (** ***n*** **, %)**				
Highly and moderately differentiated	45 (55.56%)	30 (36.59%)	5.903	0.015
Low differentiation	36 (44.44%)	52 (63.41%)		
**Lymph node metastasis (** ***n*** **, %)**				
Yes	34 (41.98%)	42 (51.22%)	1.399	0.237
No	47 (58.02%)	40 (48.78%)		
**Distant metastasis (** ***n*** **, %)**				
Yes	35 (43.21%)	34 (41.46%)	0.051	0.822
No	46 (56.79%)	48 (58.54%)		

Data are presented as *n* (%) or mean ± standard deviation. Categorical variables were compared using the Chi-square test. Continuous variables were compared using the independent samples *t*-test. *P* values were derived from two-sided tests, and a *P* value of <0.05 was considered statistically significant.

**Table 4:** Relationship between serum SAA levels and clinical pathological characteristics in PC patients

	Low SAA (*n* = 81)	High SAA (*n* = 82)	*t*/*χ* ^2^	*P*
**Age (years)**	60.33 ± 7.39	59.37 ± 6.72	0.874	0.383
**Gender (** ***n*** **, %)**				
Male	42 (51.85%)	49 (59.76%)	1.032	0.310
Female	39 (48.15%)	33 (40.24%)		
**Drinking history (** ***n*** **, %)**	35 (43.21%)	35 (42.68%)	0.005	0.946
**Smoking history (** ***n*** **, %)**	27 (33.33%)	30 (36.59%)	0.190	0.663
**Tumor location (** ***n*** **, %)**				
Head	55 (67.90%)	55 (67.07%)	0.013	0.910
Body and tail	26 (32.10%)	27 (32.93%)		
**TNM stage (** ***n*** **, %)**				
Stage I–II	41 (50.62%)	25 (30.49%)	6.852	0.009
Stage III–ⅠV	40 (49.38%)	57 (69.51%)		
**Degree of differentiation (** ***n*** **, %)**				
Highly and moderately differentiated	41 (50.62%)	34 (41.46%)	1.375	0.241
Low differentiation	40 (49.38%)	48 (58.54%)		
**Lymph node metastasis (** ***n*** **, %)**				
Yes	36 (44.44%)	40 (48.78%)	0.308	0.579
No	45 (55.56%)	42 (51.22%)		
**Distant metastasis (** ***n*** **, %)**				
Yes	28 (34.57%)	41 (50.00%)	3.975	0.046
No	53 (65.43%)	41 (50.00%)		

Data are presented as *n* (%) or mean ± standard deviation. Categorical variables were compared using the Chi-square test. Continuous variables were compared using the independent samples *t*-test. *P* values were derived from two-sided tests, and a *P* value of <0.05 was considered statistically significant.

### 3.4 Univariate and multivariate Cox regression analysis of prognostic factors in PC patients

Cox regression analysis was performed with follow-up time as the time variable and prognosis (survival = 0, deceased = 1) as the dependent variable, including the clinical and pathological variables in Table 1 as independent variables (Table 5). Univariate analysis showed that TNM stage, differentiation grade, lymph node metastasis, distant metastasis, and serum CA50, CA242, and SAA levels were significantly associated with PC prognosis (all *P* < 0.05). Multivariate analysis further identified advanced TNM stage (hazard ratio [HR] = 1.499, 95% confidence interval [CI] = 1.003–2.238, *P* = 0.048), distant metastasis (HR = 1.693, 95% CI = 1.157–2.478, *P* = 0.007), high CA50 (HR = 1.041, 95% CI = 1.019–1.064, *P* < 0.001), high CA242 (HR = 1.044, 95% CI = 1.018–1.070, *P* < 0.001), and high SAA (HR = 1.096, 95% CI = 1.044–1.151, *P* < 0.001) as independent risk factors for poor prognosis.

**Table 5:** Univariate and multivariate Cox regression analysis of prognostic factors in PC patients

Variable	Univariable		Multivariable	
	HR (95% CI)	*P*	HR (95% CI)	*P*
Age	1.014 (0.989–1.041)	0.278	—	—
Gender	0.991 (0.691–1.050)	0.963	—	—
Drinking history	1.200 (0.836–1.724)	0.322	—	—
Smoking history	1.020 (0.702–1.482)	0.917	—	—
Tumor location	1.097 (0.747–1.612)	0.637	—	—
TNM stage	1.853 (1.265–2.715)	0.002	1.499 (1.003–2.238)	0.048
Degree of differentiation	1.524 (1.054–2.203)	0.025	1.289 (0.881–1.886)	0.192
Lymph node metastasis	1.499 (1.047–2.147)	0.027	1.354 (0.942–1.945)	0.101
Distant metastasis	1.830 (1.277–2.624)	0.001	1.693 (1.157–2.478)	0.007
CA50	1.073 (1.052–1.095)	<0.001	1.041 (1.019–1.064)	<0.001
CA242	1.077 (1.052–1.102)	<0.001	1.044 (1.018–1.070)	<0.001
SAA	1.152 (1.103–1.203)	<0.001	1.096 (1.044–1.151)	<0.001

### 3.5 Combined detection of serum CA50, CA242, and SAA can assist in predicting the prognosis of PC patients

**Figure 2:**
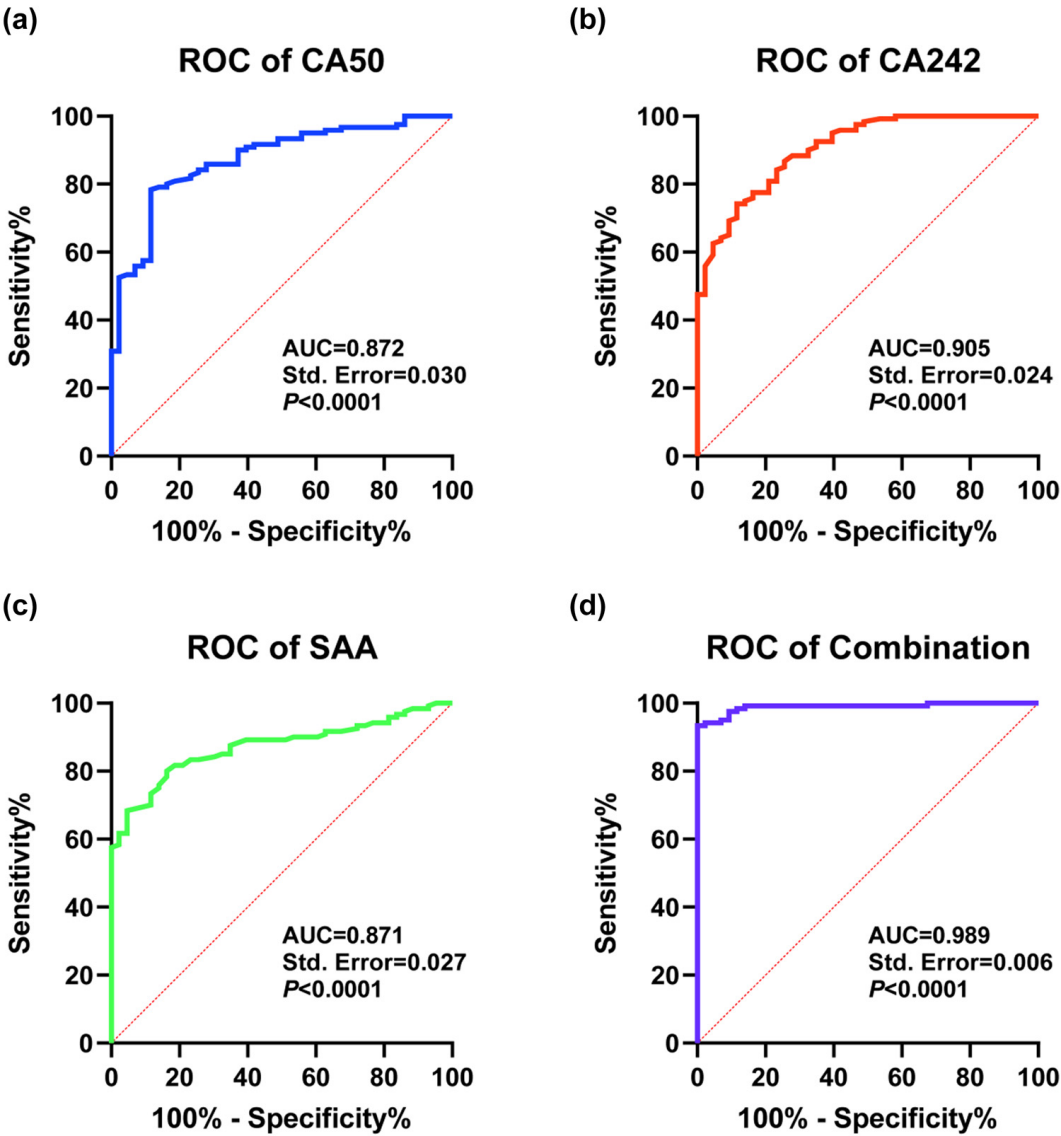
Analysis of the value of serum CA50, CA242, SAA, and combined testing in predicting poor prognosis in PC patients. ROC curves for (a) CA50 (*n* = 163), (b) CA242 (*n* = 163), (c) SAA (*n* = 163), and (d) the combination of CA50, CA242, and SAA (*n* = 163) in predicting poor prognosis (death).

ROC curve analysis was used to evaluate the prognostic predictive value of the three markers (Table 6, Figure 2). The combined detection of CA50, CA242, and SAA showed the highest AUC (0.989) with a sensitivity of 93.33% and a specificity of 100%, which was significantly superior to single-marker detection (CA50: AUC = 0.872, sensitivity = 78.33%, specificity = 88.37%; CA242: AUC = 0.905, sensitivity = 74.17%, specificity = 88.37%; SAA: AUC = 0.871, sensitivity = 80.00%, specificity = 83.72%). A comparative analysis of AUC using MedCalc demonstrated that the combined detection of serum CA50, CA242, and SAA significantly outperformed the individual detection of serum CA50 (*P* < 0.0001), CA242 (*P* = 0.0002), or SAA (*P* < 0.0001) in predicting poor prognosis in PC patients. The above results indicate that the combined detection of serum CA50, CA242, and SAA has good auxiliary predictive value for poor prognosis in PC patients.

**Table 6:** ROC curve analysis of the predictive value of serum CA50, CA242, and SAA levels for poor prognosis in PC patients

Indicator	AUC	SE	95% CI	Cut-off value	Sensitivity (%)	Specificity (%)
CA50	0.872	0.030	0.814–0.930	53.85	78.33	88.37
CA242	0.905	0.024	0.857–0.953	37.85	74.17	88.37
SAA	0.871	0.027	0.818–0.924	18.10	80.00	83.72
Combination	0.989	0.007	0.976–1.000	0.84	93.33	100.00
CA50–combination	*P* < 0.0001					
CA242–combination	*P* = 0.0002					
SAA–combination	*P* < 0.0001					

AUC, area under the ROC curve; SE, standard error; ROC curve analysis was used to assess predictive value, and MedCalc software was used for AUC comparison. All tests were two-tailed, and *P* < 0.05 was considered statistically significant.

### 3.6 Elevated CA50, CA242, and SAA levels increase the risk of death in PC patients

**Figure 3:**
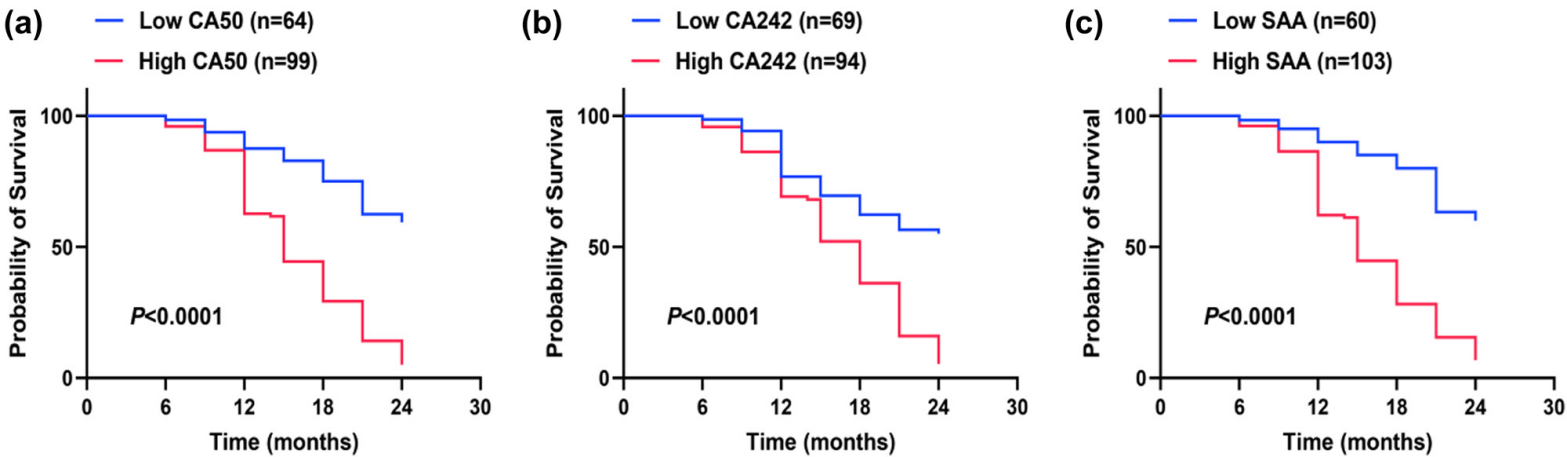
Kaplan–Meier survival curves for PC patients with different serum CA50, CA242, and SAA levels. (a) Kaplan–Meier curve for CA50 (low, *n* = 64; high, *n* = 99); (b) Kaplan–Meier curve for CA242 (low, *n* = 69; high, *n* = 94); and (c) Kaplan–Meier curve for SAA (low, *n* = 60; high, *n* = 103). The curves of the high-expression groups are shifted to the left compared with the low-expression groups. Log-rank test was used for comparison, and *P* < 0.0001 for all.

To further explore the association between serum CA50, CA242, and SAA levels and PC prognosis, PC patients were divided into high- and low-expression groups using ROC cutoff values (CA50: 53.85 U/mL; CA242: 37.85 U/mL; SAA: 18.10 mg/L) (Table 7 and Figure 3). The 2-year mortality rates were significantly higher in the high-expression groups of CA50 (78.33%), CA242 (74.17%), and SAA (80.00%) than in the corresponding low-expression groups (21.67, 25.83, and 20.00%, all *P* < 0.001). Kaplan–Meier curves (log-rank test) showed left-shifted curves in the high-expression groups of the three markers, indicating a significantly higher death risk (all *P* < 0.0001). These results indicate that patients with high expression of CA50, CA242, and SAA have a higher risk of death.

**Table 7:** Relationship between serum CA50, CA242, and SAA levels and prognosis in PC patients

	Survival	Death	*χ* ^2^	*P*
Low CA50 (*n* = 64)	38 (88.37%)	26 (21.67%)	59.071	<0.001
High CA50 (*n* = 99)	5 (11.63%)	94 (78.33%)		
Low CA242 (*n* = 69)	38 (88.37%)	31 (25.83%)	50.721	<0.001
High CA242 (*n* = 94)	5 (11.63%)	89 (74.17%)		
Low SAA (*n* = 60)	36 (83.72%)	24 (20.00%)	55.261	<0.001
High SAA (*n* = 103)	7 (16.28%)	96 (80.00%)		

## 4 Discussion

PC is one of the most lethal digestive tract malignancies worldwide, characterized by high malignancy, strong invasiveness, and poor prognosis [24]. Due to its insidious onset and lack of obvious clinical symptoms in the early stages, most patients are diagnosed when the tumor has already invaded surrounding tissues or metastasized to distant sites, leading to poor treatment outcomes [25]. In this context, tumor markers, with their non-invasive and convenient advantages, have demonstrated significant value in the early diagnosis, disease monitoring, and prognosis assessment of PC [2,26]. Therefore, identifying biological markers closely associated with the occurrence and development of PC is of great significance for improving cure rates and patient prognosis.

Although CA19-9 is the most widely used biomarker in the diagnosis and prognosis assessment of PC, its clear clinical limitations – especially the lack of specificity and the diagnostic blind spot in Lewis antigen-negative patients – have become a significant bottleneck in clinical practice. This is precisely the core motivation for exploring supplementary biomarkers such as CA50, CA242, and SAA. In terms of specificity, the diagnostic efficacy of CA19-9 is limited by the interference of benign diseases: in benign pancreatic and biliary diseases such as chronic pancreatitis and cholangitis, CA19-9 often shows non-specific elevation. Relevant studies have shown that its AUC for predicting PC is only 0.88, and the specificity is as low as 0.81 [7], which can easily lead to overdiagnosis or misdiagnosis. The more fundamental limitation stems from the biological expression mechanism of CA19-9: approximately 5–10% of PC patients have a Lewis antigen-negative phenotype and cannot synthesize CA19-9, resulting in a significant risk of false negatives in this population [27]. Not only is early diagnosis difficult, but there is also a lack of effective tools for prognosis assessment.

Accumulating evidence underscores the prognostic and predictive value of systemic inflammatory, nutritional, and biochemical markers in oncology. The host’s nutritional and metabolic status is increasingly recognized as a critical determinant of cancer progression and outcomes [28,29]. This is complemented by the established utility of specific markers such as serum calcium in CRC and the modified Glasgow prognostic score in breast cancer [30,31]. The predictive accuracy is often enhanced by multi-parameter approaches, exemplified by combinations like the NP/LHb ratio with absolute monocyte count or integrating nutritional and inflammation-related biomarkers such as the Onodera prognostic nutritional index [32,33]. Furthermore, recent investigations into pan-immune-inflammatory values and the albumin-to-globulin ratio in CRC highlight the superior prognostic power achieved by integrating tumor markers with immune-inflammatory indicators [34]. In the field of PC, the logic of “multiple indicators working together to enhance evaluation efficiency” has been further validated – the combined detection of serum D-dimer, CA19-9, and CT imaging features demonstrates significantly better diagnostic accuracy than using individual markers alone [35]. This finding is highly consistent with our conclusion that “the combined detection of CA50, CA242, and SAA optimizes the assessment of pancreatic cancer prognosis.” Together, these findings reinforce the applicability and superiority of using combined biomarkers over single indicators in the diagnosis and treatment of PC.

Our study aligns with this evolving paradigm. We hypothesized that a panel combining traditional carbohydrate antigens (CA50 and CA242) with a key acute-phase inflammatory reactant (SAA) would provide a more robust prognostic tool for PC. The development of our CA50–CA242–SAA composite marker was motivated by the need to capture both the tumor biologic activity and the host systemic inflammatory and nutritional response, which are both critical drivers of cancer progression.

In this study, serum CA50, CA242, and SAA levels in the deceased group were significantly higher than those in the survival group. From the perspective of tumor biological behavior, later TNM stage, lower differentiation, and the presence of distant metastasis all indicate higher tumor malignancy and poorer prognosis. The elevated levels of CA50, CA242, and SAA are associated with these clinical pathological features, suggesting that these three markers may be involved in the occurrence and development of PC. Specifically, high expression of CA50 and SAA was associated with higher rates of distant metastasis, while high expression of CA242 was associated with lower tumor differentiation. Among these, CA50 is a high-molecular-weight glycoprotein antigen with important clinical value in the screening and diagnosis of gastrointestinal malignant tumors (particularly PC and CRC) [36]. Previous studies have shown that CA50 is highly expressed in PC tissues, and its serum levels can serve as an auxiliary indicator for the early diagnosis of PDAC [12]. This study further found that high expression of CA50 is closely associated with TNM stage and distant metastasis, suggesting that it may play a role in tumor invasion. CA242, as a sialic acid carbohydrate, exhibits higher specificity compared to other markers (such as CA19-9) in the diagnosis of PC [17]. In this study, high expression of CA242 was associated with TNM stage and poor tumor differentiation, further validating its potential value in assessing tumor malignancy. Additionally, the gallbladder and pancreas share an embryonic origin, and their malignant tumors may share common tumor antigens. Previous studies have indicated that CA242 is a promising tumor marker in gallbladder cancer, with diagnostic performance superior to carcinoembryonic antigen (CEA) and CA19-9 [37], suggesting its potential for broad application across various gastrointestinal tumors. SAA, as an acute-phase reactant protein, plays a key role in chronic inflammation and tumor progression. This study found that high SAA expression is closely associated with TNM stage and distant metastasis, consistent with previous reports: SAA levels are not only associated with clinical stage and distant metastasis in esophageal squamous cell carcinoma but also significantly correlated with overall survival [38]. Research indicates that SAA may influence tumor initiation and progression through activation of transcription factors and the nuclear factor-kappa B (NF-κB) pathway [39,40]. Specifically, SAA can bind to receptors on the surface of tumor cells and tumor-associated macrophages, such as TLR4, triggering phosphorylation and nuclear translocation of NF-κB [41]. Activated NF-κB enhances the transcription of pro-inflammatory cytokines, including interleukin (IL)-1β (IL-1β), IL-6, and tumor necrosis factor-α [42,43]. These cytokines not only promote the proliferation and epithelial–mesenchymal transition (EMT) of PC cells, enhancing their invasive and metastatic capabilities, but also recruit immunosuppressive cells into the tumor microenvironment, thereby suppressing anti-tumor immune responses [44,45]. For example, silencing the SAA1 gene in PANC-1 cells using SAA1 siRNA significantly reduced their migratory/invasive abilities, chemoresistance, and EMT features [46]. These findings suggest that SAA can drive PC progression via the NF-κB–cytokine axis, highlighting its potential as a therapeutic target for PC intervention.

Cox regression analysis results showed that advanced TNM stage, more distant metastases, and high serum CA50, CA242, and SAA expression were all independent risk factors for poor prognosis in PC patients. Kaplan–Meier curves further indicated that patients with high expression of these three markers had a significantly increased risk of death, with survival curves shifted significantly to the left. This finding provides new biomarkers for prognosis assessment in PC. From a clinical application perspective, CA50, CA242, and SAA testing offers advantages such as simplicity, reproducibility, and non-invasiveness, providing clinicians with more accurate prognostic information to aid in the development of individualized treatment strategies [47]. For example, patients with high expression of CA50, CA242, and SAA may require more aggressive treatment regimens, such as intensive chemotherapy or targeted therapy, to improve survival rates.

ROC curve analysis results showed that the combined detection of serum CA50, CA242, and SAA significantly outperformed single-marker detection in predicting poor prognosis in PC patients, with an AUC value as high as 0.989, a sensitivity of 93.33%, and a specificity of 100.00%. This study found that CA50, CA242, and SAA exhibited higher specificity in prognostic stratification (CA50 and CA242 both at 88.37%, SAA at 83.72%), effectively reducing interference from benign diseases and providing a more reliable basis for risk stratification. Based on these findings, the excellent prognostic performance of the CA50/CA242/SAA combination (AUC = 0.989) should not be interpreted as an isolated result, but rather as strong preliminary evidence of its potential to complement CA19-9: on the one hand, in difficult cases where CA19-9 lacks specificity, this combination can provide higher discriminative accuracy; on the other hand, it expands the scope of prognostic assessment to include the high-risk Lewis antigen-negative population, thereby avoiding missed diagnoses and misjudgments. These results indicate that the combined use of multiple tumor markers can significantly improve the accuracy and reliability of predictions. This conclusion aligns with the theoretical advantages of multi-marker combination strategies: in the diagnosis of PC, the use of combined detection methods can enhance diagnostic specificity [48], and this study further expands the application scope of this combined detection strategy, confirming its significant value in assessing the prognosis of PC patients. The potential mechanism may lie in the fact that PC is a highly heterogeneous tumor, and a single marker can only reflect certain aspects of the tumor, while combined detection can comprehensively reflect multiple biological processes such as tumor proliferation, differentiation, invasion, metastasis, and inflammatory response. For example, CA50 and CA242 primarily reflect the surface characteristics and differentiation degree of tumor cells [49], while SAA reflects the inflammatory state in the tumor microenvironment [19]. The combination of these three markers can provide a more comprehensive assessment of tumor malignancy and prognosis.

In clinical practice, the combined detection of these three markers can provide stronger support for the early diagnosis, disease monitoring, and prognosis assessment of PC. For example, in PC screening, combined detection can improve the early diagnosis rate and avoid missed or misdiagnoses; during treatment, dynamic monitoring of changes in these three markers can promptly assess treatment efficacy and adjust treatment plans; in prognosis assessment, combined testing can more accurately predict patient survival outcomes, providing more valuable information for patients and their families.

This study has several limitations that need to be clearly acknowledged. First, as a single-center retrospective study, the design itself may introduce selection bias. The baseline characteristics and treatment strategies of the included patients may not adequately represent the broader population of PC patients, limiting the generalizability of the findings. Although the statistical power analysis suggests that the sample size (*N* = 163) is sufficient to detect moderate effect differences, the relatively small size of the survival subgroup (*n* = 43) may still affect the stability of the multivariate models. Second, this study lacks both internal and external validation. Although the combination model of CA50, CA242, and SAA demonstrated excellent discriminative performance (AUC = 0.989), its evaluation was based solely on a single cohort without cross-validation or independent cohort validation. This increases the risk of overfitting and may lead to an overestimation of its true predictive performance. Therefore, the current results should be considered preliminary and hypothesis-generating. Third, as an acute-phase reactant, SAA levels are susceptible to interference from nonspecific inflammation. Although patients with comorbid cancers or major organ dysfunction were excluded, potential confounding factors such as concurrent infection and autoimmune status – related inflammatory influences – were not systematically adjusted for, which may lead to an overestimation of the prognostic value of SAA specifically related to cancer itself. Finally, this study focused on clinical relevance exploration and did not conduct in-depth mechanistic research, thus failing to elucidate the specific biological pathways through which these biomarkers may contribute to PC progression. To address the above limitations, future studies should include large-scale, multicenter, prospective designs, recruiting diverse patient populations from different geographic and clinical backgrounds to enhance representativeness. All biomarkers should be measured prospectively using standardized protocols. Additionally, we will prioritize the use of cross-validation (e.g., *k*-fold cross-validation) and external validation in multicenter cohorts in subsequent studies to further confirm the robustness and clinical applicability of the model. Following this, the prognostic performance of the biomarker combination should be validated in independent cohorts and directly compared with established markers such as CA19-9, with particular attention to the Lewis-negative subgroup, to clarify its incremental prognostic value. Inflammatory markers should also be prospectively collected to control for confounding bias, and basic experimental studies should be conducted to explore the underlying molecular mechanisms.

In summary, this study demonstrates that serum levels of CA50, CA242, and SAA are closely associated with the clinical pathological characteristics and prognosis of PC patients. The combined detection of these three markers can serve as an effective auxiliary indicator for assessing poor prognosis in PC, providing new insights and methods for the diagnosis, treatment, and prognostic assessment of PC.
